# Area 8A within the Posterior Middle Frontal Gyrus Underlies Cognitive Selection between Competing Visual Targets

**DOI:** 10.1523/ENEURO.0102-20.2020

**Published:** 2020-09-04

**Authors:** Jürgen Germann, Michael Petrides

**Affiliations:** 1 Cognitive Neuroscience Unit, Montreal Neurological Institute, McGill University, Montreal, Quebec H3A 2B4, Canada; 2 McConnell Brain Imaging Centre, Montreal Neurological Institute, McGill University, Montreal, Quebec H3A 2B4, Canada

**Keywords:** cognitive control, cytoarchitectonic area 8A, dorsal attention network, dorsolateral prefrontal cortex, higher order attention, visual selection

## Abstract

There are several distinct areas in the granular part of the lateral frontal cortex, and these areas provide high-level regulation of cognitive processing. Lesions of the dorsolateral frontal cortex that include area 8A in the human brain and lesions restricted to area 8A in the macaque monkey have demonstrated impairments in tasks requiring selection between visual targets based on rules, such as conditional if/then rules. These same subjects show no impairment in the ability to discriminate between visual stimuli nor in the ability to learn selection rules in general. Area 8A can be considered as a key area for the top-down control of attentional selection. The present functional neuroimaging study demonstrates that activity in area 8A that lies on the posterior part of the middle frontal gyrus underlies the trial-to-trial selection between competing visual targets based on previously acquired conditional rules. Critically, the activity of area 8A could clearly be dissociated from activity related to the performance of eye movements per se that lies posterior to it. Thus, area 8A with its rich corticocortical connections with the posterior parietal region involved in spatial processing and the multisensory temporal cortex appears to be the key prefrontal area for the higher order selection between competing stimuli in the environment, most likely by the allocation of attention.

## Significance Statement

Dorsolateral frontal lesions that include area 8A in the human brain and selective lesions to area 8A in monkeys impair the ability to select between competing visual targets based on learned rules, such as if/then conditional rules. There is no impairment in the ability to discriminate between visual stimuli and to learn to select in general after such lesions. Area 8A can be considered as a key area for the top-down regulation of attentional selection. The present functional neuroimaging experiment demonstrates that the specific cognitive control process performed by this area is the higher order selection between competing stimuli in the environment based on internal knowledge (e.g., rules), presumably by the allocation of attention.

## Introduction

The various areas of the lateral prefrontal cortex are known to be critically important for cognitive control. Patients with lesions to these areas are impaired when performance depends on the adequate application of various control processes (Stuss and Knight, 2002; Petrides, 2005; Shallice et al., 2008). Lesions in the dorsolateral frontal cortex of the human brain have been shown to impair performance on conditional associative tasks (Petrides, 1985a), and macaque monkey studies have demonstrated that the critical area lies in the periarcuate region in the caudal dorsolateral frontal cortex (Petrides, 1985b, 1986, 1987). These tasks require the trial-by-trial selection of specific responses/stimuli from a set of alternatives based on conditional rules: if instructional stimulus A, select response/visual stimulus X, but if instructional stimulus B, select response/visual stimulus Y, etc. The macaque monkey studies have shown that lesions restricted to area 8A impair the selection between alternative visual stimuli (Petrides, 1987). Caudal dorsolateral frontal lesions that include area 8A do not impair visual discrimination or the process of selecting per se: It is the use of the appropriate conditional rules for the trial-to-trial flexible selection between the alternative visual stimuli that is impaired (Petrides, 1985a,b, 1986, 1987).

**Figure 1.**
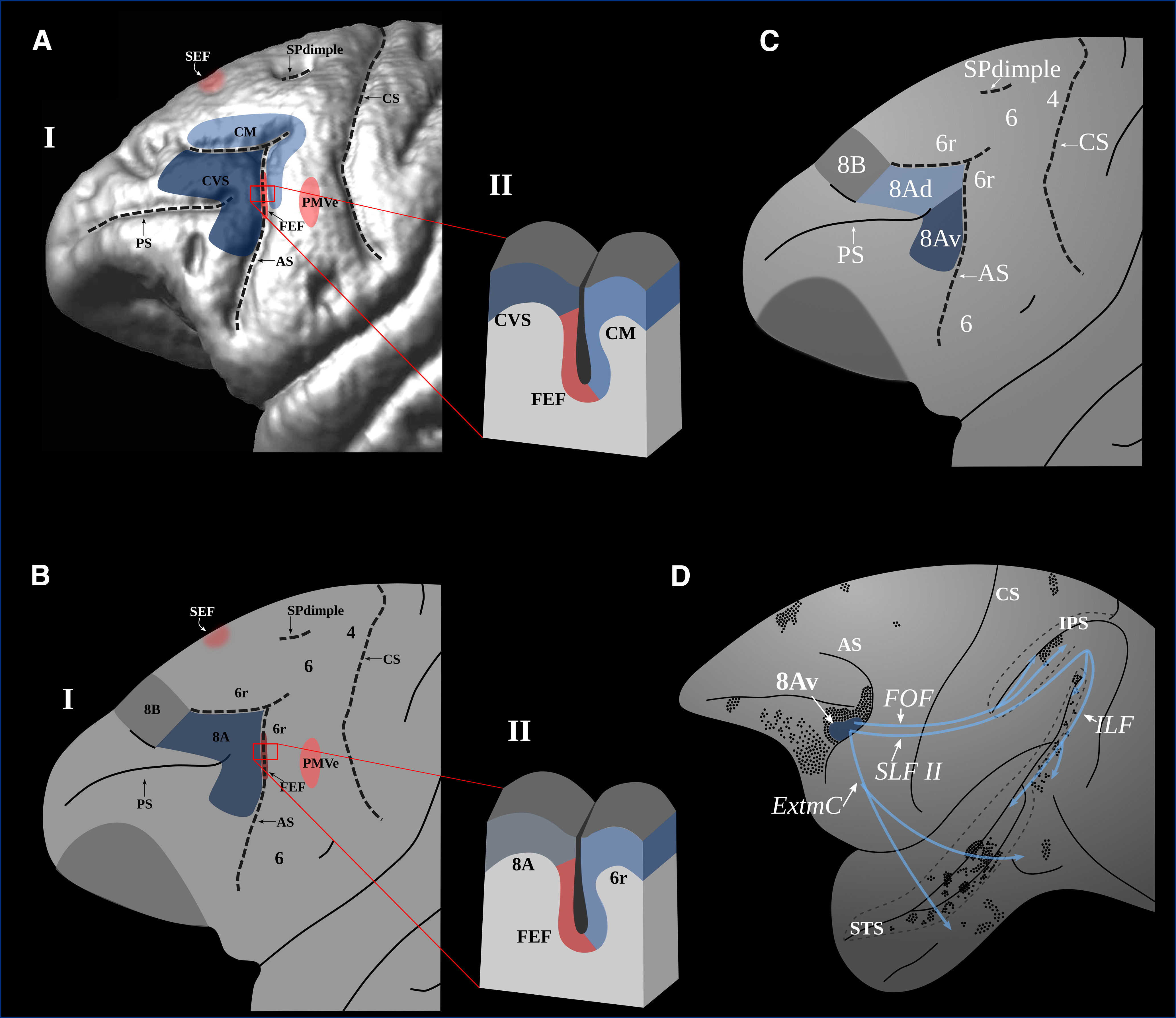
The arrangement of relevant areas in the macaque frontal lobe. 3-D reconstruction of the magnetic resonance image of a macaque monkey brain (***A***) and a schematic drawing of the frontal region (***B***); the three eye movement-related areas are in shades of red: the FEF, SEF, and PMVe (Bruce et al., 1985; Bruce and Goldberg, 1985; Fujii et al., 1998). In shaded area posterior to the arcuate sulcus outlines, the areas where lesions impair monkey performance on conditional motor selection tasks (CM), i.e., anterior area 6 in the posterior bank of the arcuate sulcus (Halsband and Passingham, 1982; Petrides, 1987) and the area lesions of which impair the conditional selection of visual stimuli (CVS), i.e., area 8A (Petrides, 1987). ***A***, ***BII***, Section through the arcuate sulcus to show the locus of the microstimulation elicited eye movements, i.e., the FEF (Bruce et al., 1985; Bruce and Goldberg, 1985). This region lies immediately posterior to granular area 8A (Stanton et al., 1989; Tehovnik et al., 2000; Gerbella et al., 2007). ***C***, Area 8A can be divided, based on the characteristic corticocortical connectivity pattern, into a ventral (i.e., area 8Av) and a dorsal part (i.e., area 8Ad). The ventral part of area 8Av is connected to higher-order visual occipital and temporal areas (Petrides and Pandya, 1988, 1999, 2006). ***D***, Corticocortical connectivity pattern of area 8Av showing cortical terminations and association fiber pathways (adapted from Petrides and Pandya, 2006). AS: arcuate sulcus; CM: conditional motor selection; CS: central sulcus; CVS: conditional visual selection; ExtmC: extreme capsule; FEF: frontal eye field; FOF: fronto-occipital fasciculus; ILF: inferior longitudinal fasciculus; IPS: intra-parietal sulcus; PMVe: ventral premotor eye area; PS: principal sulcus; SEF: supplementary eye field; SPdimple: superior precentral dimple; SLF II: superior longitudinal fasciculus II; STS: superior temporal sulcus.

In the earlier neuroscience literature, the term “area 8” was often used to refer to the frontal eye field (FEF). This view originated from early research by Ferrier showing that electrical stimulation of the periarcuate region in monkeys induced eye movements (Ferrier, 1875). However, subsequent research using electrical microstimulation demonstrated that the region from which eye movements can be elicited lies within the arcuate sulcus (Bruce et al., 1985; Bruce and Goldberg, 1985; Tehovnik et al., 2000; Fig. 1*A*,*B*). Area 8A is a clearly granular prefrontal cortical region that lies anterior to the arcuate sulcus (Petrides and Pandya, 1999). These findings suggest that the FEFs may in fact lie in the transition zone between agranular premotor area 6 and 8 rather than in granular area 8A proper. Functional neuroimaging studies confirmed that the activation peaks related to eye movement per se in the human brain lie posterior to the location of granular area 8A. Eye movement-related activations were in the superior precentral sulcus (PCS) and also in the inferior PCS, the latter area probably corresponding to the ventral premotor eye area (PMVe) described by Fuji and colleagues in the monkey (Paus, 1996; Fujii et al., 1998; Amiez et al., 2006; Amiez and Petrides, 2009).

Granular prefrontal area 8A that lies clearly anterior to the FEF areas is now often considered as a key region regulating visual attention (Petrides, 1987, 2005; Corbetta and Shulman, 2002; Fox et al., 2006) and is a key component of the dorsal attention network identified in resting state functional magnetic resonance imaging (fMRI) data (Fox et al., 2006; Yeo et al., 2011; Szczepanski et al., 2013; Baldauf and Desimone, 2014). Anatomical studies indicate that area 8A can be subdivided into a dorsal part (i.e., area 8Ad) and a ventral part (i.e., area 8Av; Fig. 1*C*) based on their corticocortical connectivity pattern. Area 8Av is connected with the caudal intraparietal sulcal region and adjacent posterior inferior parietal lobule (area PG), the multisensory cortex in the superior temporal sulcus, and the visual peristriate inferior occipital and occipito-temporal region (Fig. 1*D*; Petrides, 1987; Petrides and Pandya, 1999, 2006). This granular frontal area is, therefore, connected with both the “what” and the “where” streams of visual information processing (Mishkin and Ungerleider, 1982; Mishkin et al., 1983) and is ideally situated to control the allocation of visual attention in tasks (such as the conditional associative tasks) requiring selection between competing visual stimuli from trial to trial based on the application of if/then rules (Petrides, 1987).

Based on the information considered above, namely, that (1) the eye fields lie posterior to granular area 8A; (2) that area 8Av may be involved in the top-down control of visual attention; and (3) that lesions restricted to area 8A impair the rule-based trial by trial selection between alternative visual stimuli, we hypothesize that the specific contribution of ventral area 8A is the cognitive selection between competing visual stimuli via the allocation of attention based on internal knowledge, rather than the performance of eye movements per se. To test this hypothesis, we designed the present neuroimaging study to examine functional activation related to the conditional, rule-based selection between visual stimuli and compare it to activation related to the performance of eye movements per se.

## Materials and Methods

### Experimental design

The present fMRI study tested the hypothesis that area 8A is critical for the cognitive selection of stimuli via the allocation of attention based on internal knowledge and compared the role of area 8A with that of frontal cortical areas involved in eye movement control per se. The subjects were required to perform two tasks: an experimental task in which they cognitively select, based on previously learned conditional rules, which one of four possible locations to look at; and a control task requiring the performance of identical eye movements guided by an external stimulus, i.e., a task in which no cognitive selection between alternative locations is required.

This design permitted us to assess activation related to the making of eye movements toward particular locations on a screen under two distinct conditions. In the experimental condition, the selection (on any given trial) of which one of four competing target locations to look at is determined by the subject based on a previously learned cognitive rule: select one of the four competing locations and move the eyes to it depending on the color of the instruction stimulus (if yellow, select and move the eyes to location X, but if green, select location Y, etc.). By contrast, in the control condition, a neutral instruction stimulus carries no information about which one of the four locations to look at and the eyes are directed to the location that is marked in black, i.e., the decision where to direct eye movements is determined externally by the experimenter. Thus, eye movement to one of four locations is required in both conditions, but in the experimental condition the subject is, additionally, required to make a cognitive decision based on prelearned conditional instruction cues. Comparison of activations between these two conditions and comparisons of each one of these two conditions with a simple fixation condition would allow us to determine activation related to (1) the production of eye movements per se and (2) activation related to a cognitive rule-based decision of where to direct the eyes. The aim is to examine frontal areas involved in eye movement production per se in contrast to areas involved in the higher order cognitive decision (i.e., selection) of where to look.

**Figure 2.**
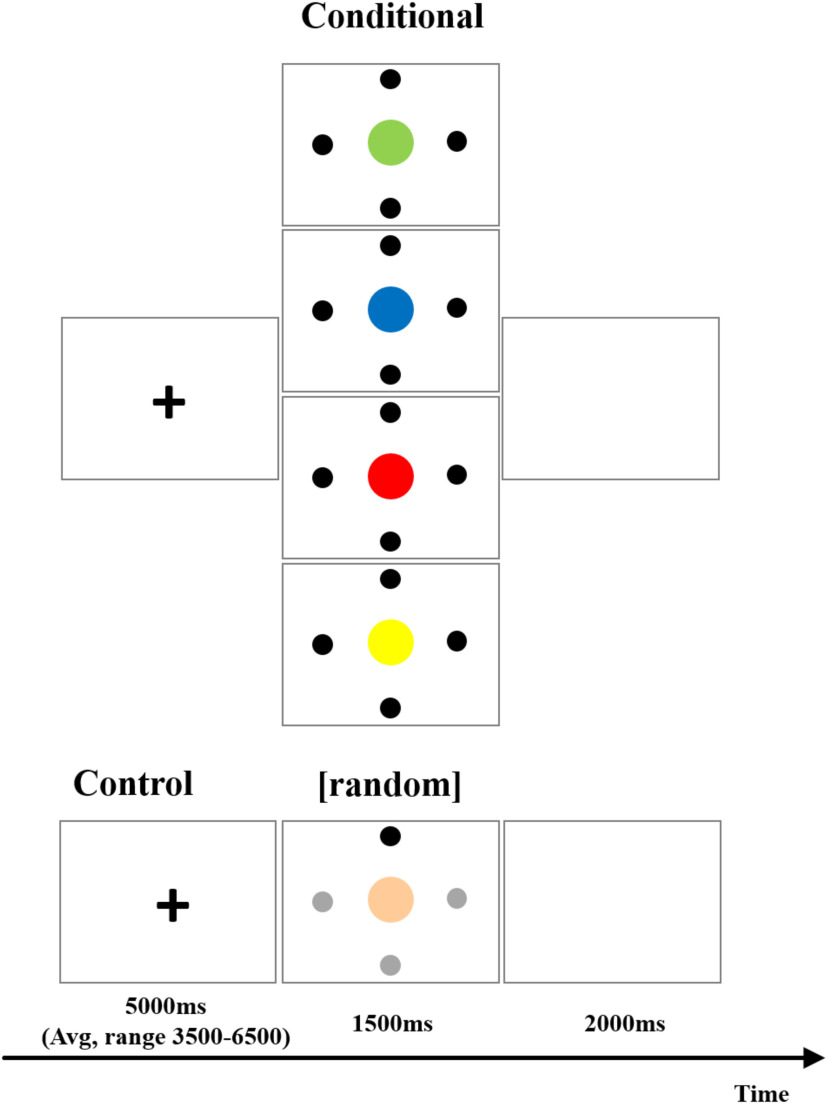
Timeline illustrating the various trials of the fMRI experiment. On the top are the four conditional trials. Subjects had learned (before scanning) which one of the black circles had to be selected based on the color of the central circle and moved their eyes to look at the appropriate black circle. In the control condition, the central color was always beige and one of the circles was black and the others gray and the subjects moved their eyes to the one black circle. The location of the black circle (as opposed to the gray ones) was determined according to a random order with all four locations appearing in black with the same frequency over one complete run. On each particular trial, control or conditional, subjects were presented with a colored circle in the center and four marked locations surrounding it and had to move their eyes to one particular location. Thus, in the control condition, the exact same looking and eye movement response was required, as in the conditional task, but the response was determined by the shading of the dot, and, therefore, the subject did not have to make a selection between alternative looking responses based on conditional rules.

The 13 participants in this fMRI experiment (seven females) were 20–30 years old (mean 24.3 years, SD 3 years). These subjects had learned to make saccadic eye movements to one of four target locations depending on the color of the circle presented in the center: green, blue, red, or yellow. The day before the fMRI experiment, each subject was placed in front of a screen and presented with images that showed a central circle surrounded by four black circles (Fig. 2). The color of the central circle provided information about which one of the four black surrounding circles should be the target of an eye movement. The four black target location circles were ∼20° from the center of the screen. On any given trial, the particular color within the central circle constituted the instruction to make a fast eye movement to one of the four black circles (Fig. 2). The stimuli were otherwise identical and the colored circle was always presented with the four black dots (Fig. 2). The subject learned (by trial and error) which one of the four locations was indicated by each color in this prescanning training session. In this session, each subject decided toward which location to move the eyes based on the particular color of the central circle (i.e., the instruction stimulus). After a few practice trials (three to four trials per target) to familiarize the subject with the task, the training session started. Now, a series of trials followed during which one of the four stimuli with the distinctly colored circle in the center was presented every 2 s, and the subject had to select one of the target locations and make an eye movement toward it. The sequence of presentation of the four color instructional stimuli surrounded by the four black circles was pseudorandom, i.e., all colors appeared with the same frequency over the course of the whole series of trials. The subject received feedback verbally from the experimenter only when a saccadic eye movement to an incorrect target was performed and that trial was repeated.

The eye movements were monitored via a mirror system and also recorded using a video camera to check for errors later at slower speed. The goal of this training session was to allow the subjects to learn the conditional rules (e.g., if the instruction stimulus is yellow, move the eyes to location X, but if the instruction stimulus is green, move the eyes to location Y, etc.; Fig. 2). The subjects learned quickly, i.e., after ∼30–40 trials, they were able to perform the task. The criterion of having learned the task during this training session was the performance of 120 consecutive trials without error. The subjects were then introduced to the control trials. Here, the arrangement was similar to the experimental one in that it was composed of a central circle surrounded by four dots. In the control condition, the instructional stimulus, i.e., the color of the central circle, was always the same, i.e., a beige color and three of the surrounding circles were now gray and only one was black (Fig. 2). Again, the colored circle and the four dots were presented at the same time. The subjects were instructed to make a saccade to the black circle. The location of the black circle was random, but balanced as each of the four locations was used the same number of times across each run. This condition which required eye movement to a particular location driven externally served as the control condition for the experimental condition which required the selection by the subject of the location to direct the eyes based on the color of the circle (instructional stimulus). We could thus compare activations in conditional eye movement selection compared with the control stimulus-driven eye movement. Also, the subjects learned to fixate at the center of the screen when a cross was presented there. Fixating on the cross was used as a baseline condition for the control stimulus-driven eye movement trials which could be compared with the fixation baseline. On 96 subsequent random trials of conditional and control stimuli, all subjects made no errors. Before the scanning session on the following day, all subjects successfully repeated another set of 96 random mixed trials (conditional and control trials) as a refresher. The large number of training trials over 2 days, i.e., performing extra training sets even after all the subjects had learned to perform the task without errors, was chosen to ensure that both the experimental and control tasks were performed effortlessly and to minimize any additional brain activity that might confound the interpretation of the results.

The subjects, now in the scanner, were instructed to perform the task as had been learned. In the scanner during each run, each one of the conditional instruction cues (one of the four instructional colors) appeared eight times mixed with an equal number of trials presenting the control stimulus (beige color) all in random order (32 conditional + 32 control trials = 64 trials in total). Each trial started with a fixation cross presented for 5 s (± random jitter of up to 1.5 s), followed by the instruction color stimulus or the control beige stimulus presented for 1.5 s. The trial ended with a blank screen (1 s; Fig. 2).

### MRI acquisition

Scanning was performed in a 1.5T Siemens Sonata MRI Scanner (Siemens). After acquiring a 1-mm isotropic T1 weighted anatomic scan, three runs of 170 images (∼10 min) each (T2* gradient echo images, 38 oblique slices, TR = 3500 ms, TE = 50 ms, 90° flip angle, matrix 38 × 64 × 64, 3.4 mm isotropic) sensitive to the blood oxygen level-dependent (BOLD) signal were acquired.

### fMRI data preprocessing

Using the third frame as reference (i.e., after discarding the first two frames), all images per run were realigned with an AFNI image registration software (Cox, 1996) and smoothed using a 6-mm full-width at half-maximal (FWHM) Gaussian kernel (mincblur; http://www.bic.mni.mcgill.ca/ServicesSoftware/MINC). The images were then transformed into MNI space (Collins et al., 1994; Cox, 1996) using the transformation derived from registering the anatomic images and the functional and anatomic data were merged for localization.

### Statistical analyses

Fmristat (Worsley et al., 2002) was used to analyze the functional data. First, fMRI data were converted to a percentage of the average signal intensity over all intracerebral voxels and slice timing correction implemented. Univariate linear modeling with correlated error was used for statistical analysis. The paradigm was an event-related design with three events (fixation, conditional selection of eye movement, stimulus-driven eye movement). The onset of the fixation was the presentation of a cross, the onset of the other two conditions was the presentation of an instruction stimulus which led to the subsequent eye movement.

The resulting *t* statistic images were thresholded using the minimum given by Bonferroni correction and random field theory. This approach takes spatial correlation of the error into account. For a single voxel within an estimated gray matter volume of 600 cm^3^ the threshold of reporting a peak as significant (*p*_corrected_ < 0.05) was *t* > 3.3 (Worsley et al., 1996, 2004).

MATLAB (The MathWorks) was used to calculate the average (across all subjects and trials) BOLD signal response curves for each condition (fixation, experimental condition, control condition) for the six peaks identified in the previous analysis. Time zero was set at presentation onset. Percentage change in BOLD relative to time 0 was calculated and plotted against time since stimulus onset.

**Figure 4.**
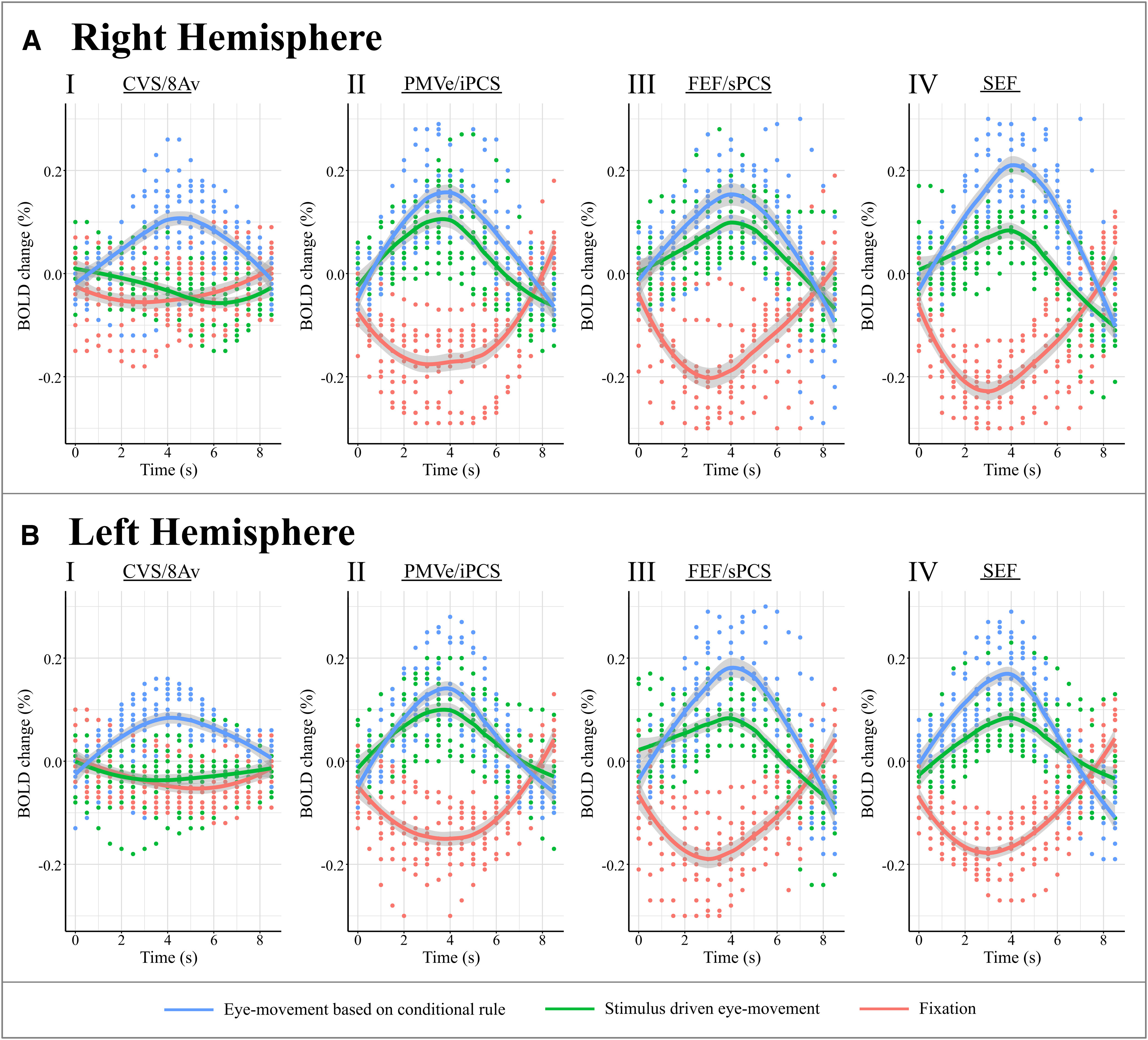
The BOLD level hemodynamic response curves reconstructed in all subjects for the four relevant peaks in each hemisphere (only peak voxel for each subject/contrast; using all trials over the 3 runs). The mean curves are plotted with standard error as shaded area for the right (***A***) and left (***B***) hemispheres. The peaks are: CVS/8Av (***I***); PMVe/iPCS (***II***); FEF/sPCS (***III***); SEF (***IV***). Zero second is event onset (presentation of the stimulus that initiates the eye-movement to a particular location). The conditional visual selection peak (CVS) in area 8Av shows changes in BOLD response *only* in the conditional selection condition in which the target is selected on the basis of an internal cognitive decision according to a known rule. It is important to note that this area -as opposed to all eye-movement areas- is not involved in moving the eyes per se (compare stimulus-driven eye movement (green) vs eye movement based on conditional rule (blue) in AI and BI). Also note that the effects are found across all subjects. CVS/8Av: Conditional Visual Selection in area 8Av in the posterior middle frontal gyrus; iPCS: inferior Precentral Sulcus eye area; PMVe: Premotor Eye-area; FEF: Frontal Eye Field area; SEF: Supplementary Eye Field area; sPCS: superior Precentral Sulcus eye area.

## Results

### fMRI peaks

**Figure 3.**
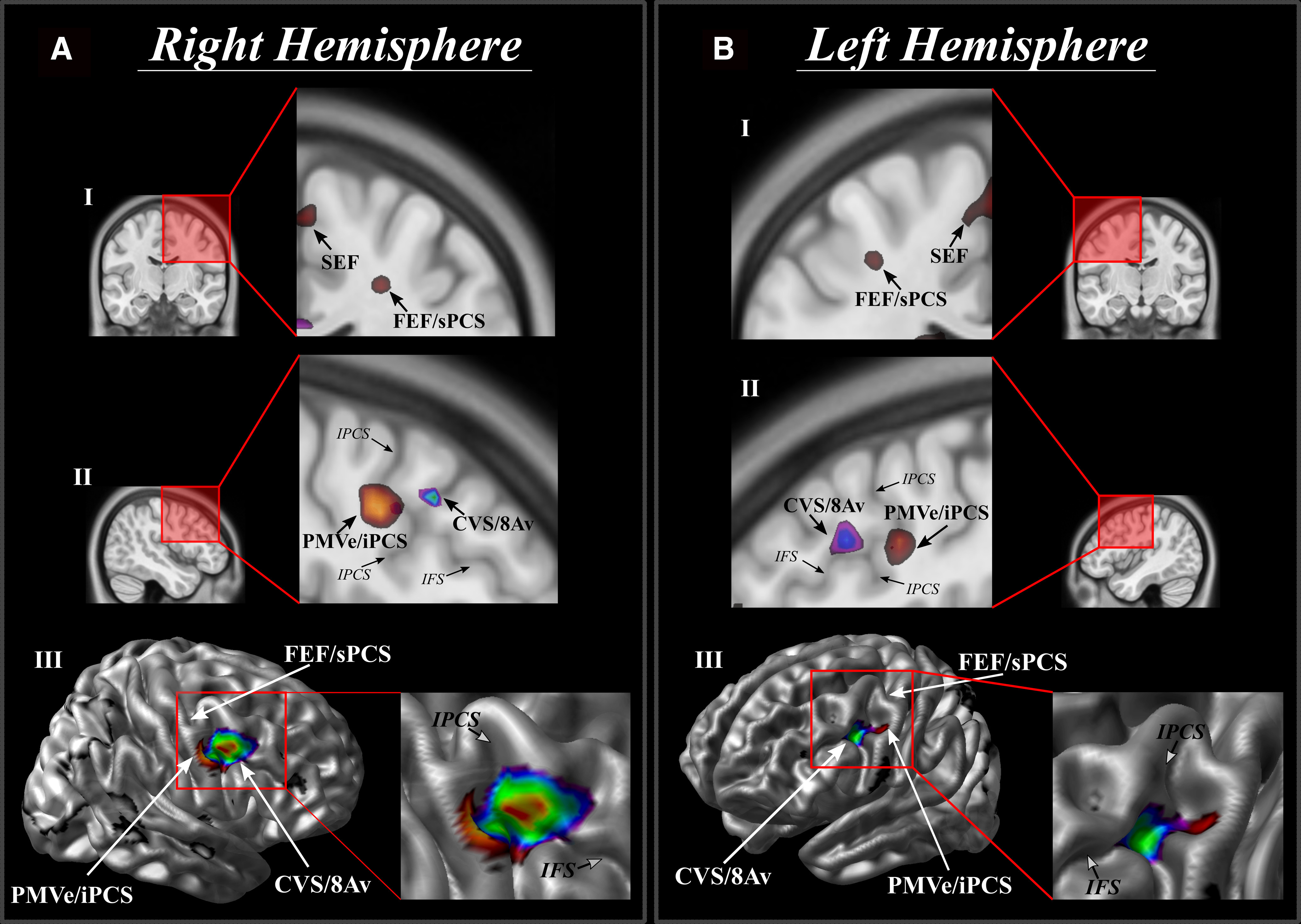
The location of the activation peaks demonstrated in the functional experiment: the conditional visual selection in area 8Av (CVS/8Av), the FEF in the superior PCS (FEF/sPCS-superior PCS eye area), the SEF on the medial frontal region and the PMVe (PMVe/iPCS-inferior PCS eye area). The peaks associated with eye movement (i.e., eye movement condition MINUS fixation) are shown in hot colors. The peak associated with the conditional eye movement looking responses MINUS control location-induced eye movement is in spectral color. ***A***, Right hemisphere. ***B***, Left hemisphere. ***I***, SEF and FEF in a coronal section. ***II***, CVS in area 8A and the PMVe in a sagittal section. ***III***, Peaks superimposed on a 3-D reconstruction of the brain in a lateral oblique angle with the enlarged area showing the region in the posteroventral part of the middle frontal gyrus at the junction of the inferior frontal sulcus (IFS) and inferior PCS (IPCS), i.e., where area 8Av is located (Petrides and Pandya, 1999; Petrides, 2019).

Compared with fixation, eye movement per se (control stimulus-driven eye movement minus fixation) is associated with three distinct bilateral activation peaks in the precentral region of the frontal lobe in both the right and left hemispheres: one peak was in the ventral ramus of the superior PCS where the FEF/sPCS peak in the human brain is known to be located (Paus, 1996; Amiez et al., 2006; Amiez and Petrides, 2009; Tark and Curtis, 2009; Leoné et al., 2014; Mackey et al., 2017). This peak was significant only in the right hemisphere (Fig. 3; Table 1). The ventral ramus of the superior PCS is known to show great interindividual variability and the relatively low t-statistics of this peak might be because of this individual variability that would limit the group analysis (Germann et al., 2005; Amiez et al., 2006; Amiez and Petrides, 2009). The other peaks related to eye movement per se were in the ventral premotor cortex (the PMVe/iPCS peak; Tark and Curtis, 2009; Leoné et al., 2014; Mackey et al., 2017) that may correspond to the ventral premotor eye movement area in the monkey (Fujii et al., 1998) and also in the dorsomedial frontal cortex, the so-called supplementary eye field (SEF; Schlag and Schlag-Rey, 1987). Similar peaks have been reported previously in other fMRI experiments investigating eye movements (Petit et al., 1997; Petit and Haxby, 1999; Parton et al., 2007). These regions in the human brain that are activated in relation to eye movements per se are also referred to as sPCS (superior PCS eye movement area) for the dorsal region (FEF) and iPCS (inferior PCS eye movement area) for the ventral one (PMVe; Tark and Curtis, 2009; Leoné et al., 2014; Mackey et al., 2017).

**Table 1:** Significant function activation peaks in area 8Av and the FEFs in the fMRI experiment

Right hemisphere					
Structure	Coordinates			*t* value	fMRI contrast
	*x*	*y*	*z*		
CVS/8Av	43	18	37	7.6*	Conditional *minus* control eye movement
PMVe/iPCS	56	4	38	7.9*	Control eye movement *minus* fixation
FEF/sPCS	26	–12	42	4.2*	Control eye movement *minus* fixation
SEF	05	–12	63	4.2*	Control eye movement *minus* fixation
Left hemisphere					
Structure	Coordinates			*t* value	fMRI contrast
	*x*	*y*	*z*		
CVS/8Av	–32	18	26	6.1*	Conditional *minus* control eye movement
PMVe/iPCS	–50	0	37	5.7*	Control eye movement *minus* fixation
FEF/sPCS	–31	–13	47	2.1	Control eye movement *minus* fixation
SEF	–01	–11	62	4.1*	Control eye movement *minus* fixation

CVS/8Av: conditional visual selection in area 8Av in the posterior middle frontal gyrus; iPCS: inferior PCS eye area; PMVe: premotor eye-area; FEF: frontal eye field area; SEF: supplementary eye field area; sPCS: superior PCS eye area; *p_cor_ < 0.05.

**Figure 5.**
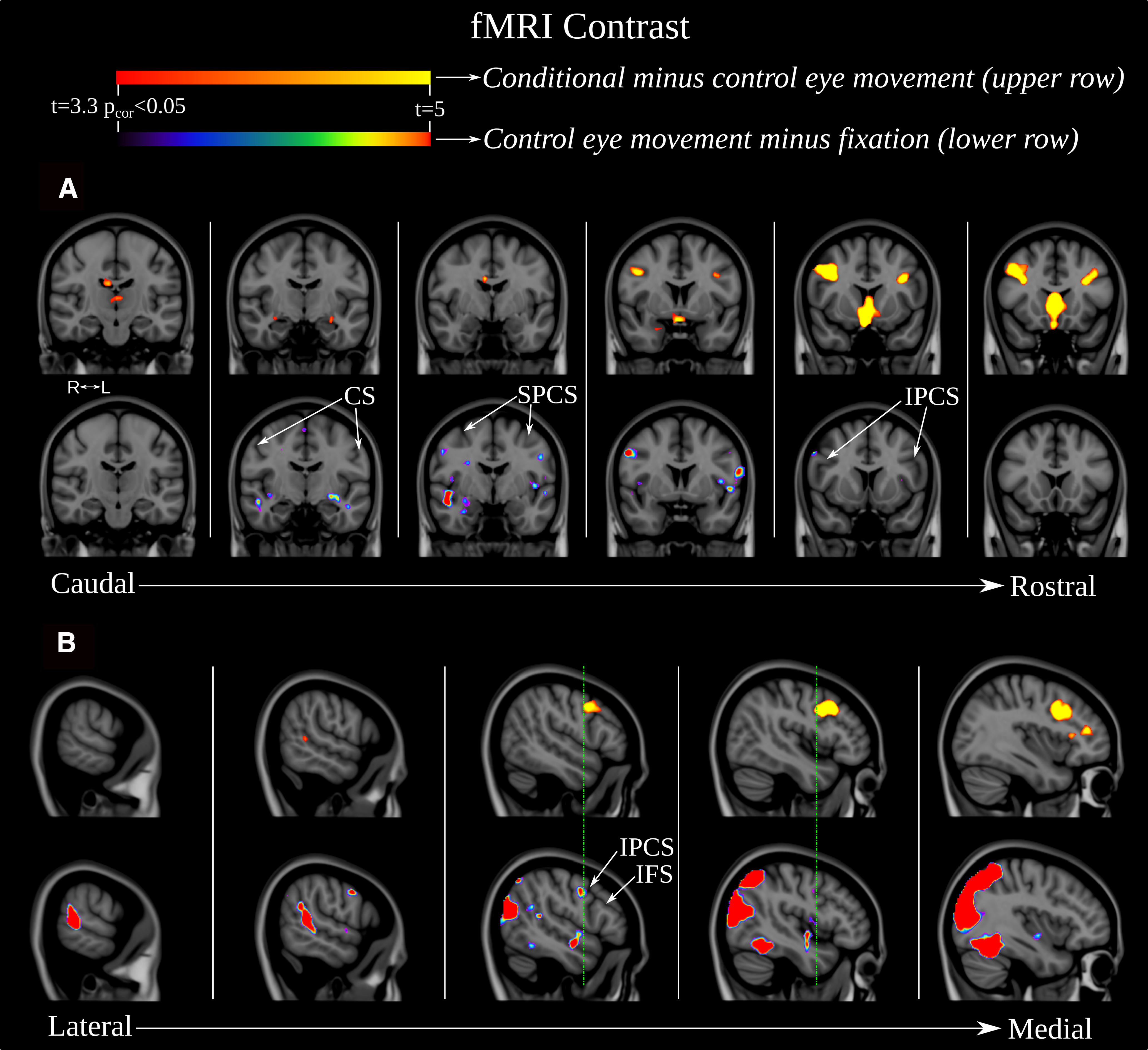
Series of coronal (***A***) an sagittal (***B***) sections illustrating in the in the top rows voxels whose activity is significant in the ‘conditional eye-movement MINUS control eye-movement’-contrast in hot colors and in the bottom rows (identical sections) voxels whose activity is significant in the ‘eye-movement condition MINUS fixation’-contrast in spectral colors. The detailed illustration shows that the significant clusters are clearly spatially distinct: activity associated with the “eye movement condition MINUS fixation” contrast is found only on the posterior bank of the inferior PCS and the precentral gyrus, whereas activity associated with the “conditional eye movement MINUS control eye movement” contrast is found only in the ventro-caudal middle frontal gyrus, rostral to the inferior PCS. The vertical green lines in ***B*** provide a reference to emphasize the separation of these peaks along the *y*-axis.

The conditional selection task (conditional selection task minus control stimulus-driven eye movement), however, reliably elicits a significant response in one region located a considerable distance anterior to the areas related to eye movement per se (Figs. 3, 4, 5; Table 1) on the middle frontal gyrus where granular area 8A is found (Petrides and Pandya, 1999). As can be seen in Table 1 and Figure 5, the PMVe/iPCS peak and cluster of significant voxels is found on the precentral gyrus (Y = 4), while the peak and the entire cluster of significant voxels associated with the conditional selection task are found quite a distance from the previous precentral peak in the ventro-caudal middle frontal gyrus (Y = 18; Fig. 5), i.e., a mean difference of 14 mm in stereotaxic space. Thus, the peaks related to the performance of eye movement per se lie on the precentral gyrus where the rostral premotor region lies, while the conditional attentional selection peak lies on the posterior middle frontal gyrus where granular area 8A lies (Petrides and Pandya, 1999). The peak, which was thus uniquely associated with the internal rule-guided cognitive decision of where to move the eyes, was located in the posterior middle frontal gyrus at Y = 18 in MNI stereotaxic brain space (Figs. 3, 5; Table 1). This area has been identified in cytoarchitectonic studies as ventral granular area 8A (Petrides, 2019; Petrides and Pandya, 1999). As expected, all four peaks are present when contrasting the conditional eye movement task with the fixation task (Fig. 4).

### BOLD response curves and location of peaks

**Figure 6.**
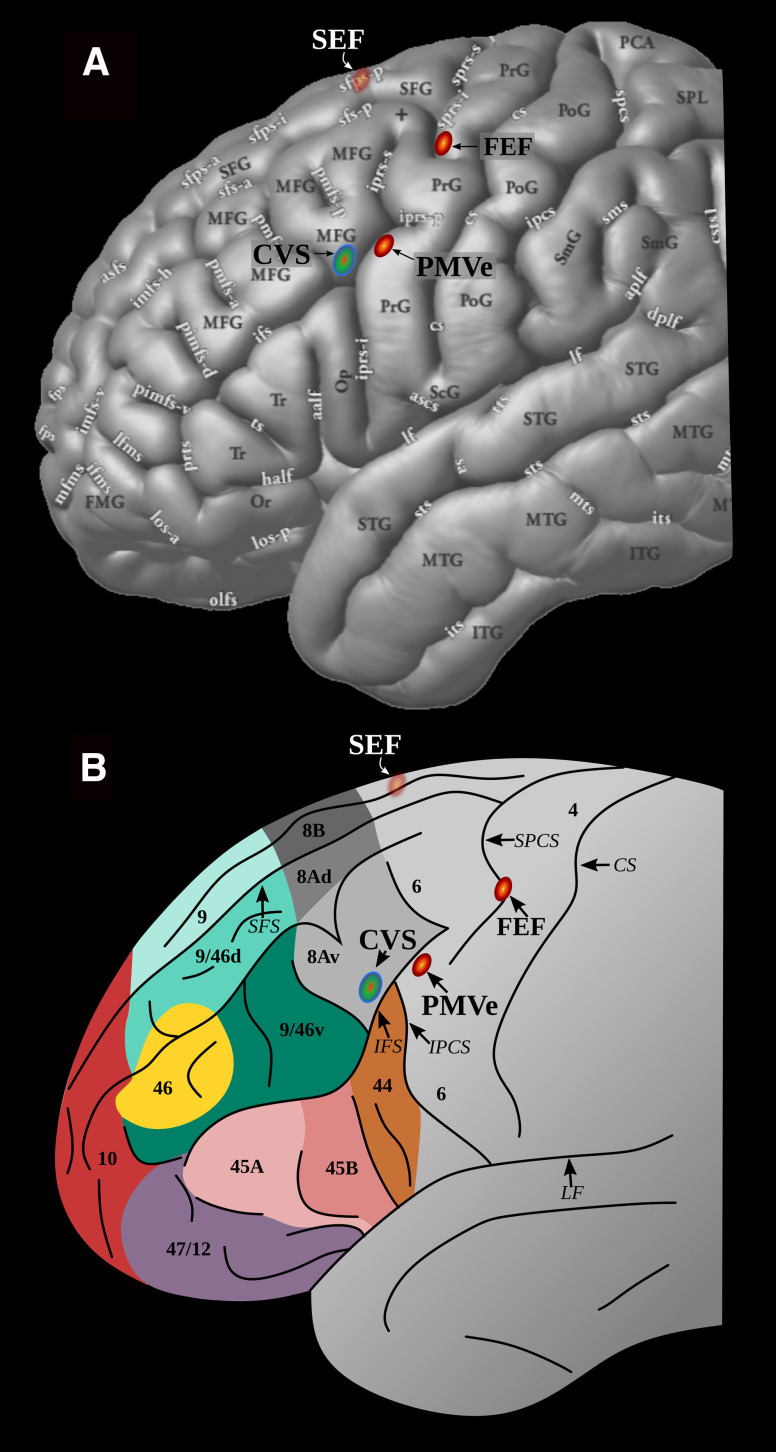
Location of the relevant functional areas in the human brain as demonstrated in the current functional neuroimaging study. The regions are comparable to those illustrated in the monkey brain in Figure 1. ***A***, 3-D reconstruction of the average MNI human brain adapted from Petrides (2019). ***B***, Outline of cytoarchitectonic areas of the human brain based on a comparative cytoarchitectonic study of macaque monkey and human frontal cortex (Petrides and Pandya, 1999). The three eye movement-related peaks identified in the present fMRI experiment are shown in red: the FEF/sPCS, SEF, and PMVe/iPCS, and the conditional visual selection (CVS) peak is shown in blue. CS: central sulcus; CVS: Conditional Visual Selection in area 8Av in the posterior middle frontal gyrus; PMVe: Premotor Eye-area; FEF: Frontal Eye Field area; LF: lateral fissure; IFS: inferior frontal sulcus; IPCS: inferior precentral sulcus; SEF: Supplementary Eye Field area; SFS: superior frontal sulcus; SPCS: superior precentral sulcus.

The reconstructed hemodynamic response function (hrf) curves for these four peak locations demonstrated that, while the FEF/sPCS, SEF and PMVe/iPCS show significant responses to all eye movements (regardless of whether they were internally guided by conditional rules or driven by the target stimulus), the response of the peak in ventral area 8A is limited to internally guided (i.e., cognitively rule-based selected) eye movements (Fig. 4). Some additional prefrontal peaks are found more rostrally probably related to the subjects validating the accuracy of their selection as they were monitoring their performance (for a complete list of all additional significant peaks, see Table 2; Petrides, 2005; Champod and Petrides, 2007; Procyk et al., 2016; Amiez and Petrides, 2018). The spatial arrangement of the four frontal peaks related to the looking response, both internally and externally guided, is very similar to the arrangement found in monkeys and is in accordance with comparative cytoarchitectonic studies (Figs. 1, 6). The results of the present fMRI study indicate that the ventral part of area 8A, namely the granular area on the middle frontal gyrus (that lies clearly anterior to the eye movement-related posterior peaks in the rostral precentral region) plays a critical role in guiding attentional selection to external locations based on internal cognitive rules (if instruction cue A is presented, look at location X, but if instruction stimulus B is presented, look at location Y, etc.).

**Table 2:** Additional peaks passing *p*_cor_ < 0.05 in the fMRI experiment

Conditional minus control eye movement			
Coordinates			*t* value
*x*	*y*	*z*	
37	40	14	5.4
0	30	13	6.7
38	26	10	4.8
2	12	–15	7.6
27	4	–25	4.1
–26	–14	–18	4.5
2	–32	35	5.5
60	–42	6	5.5
–34	–52	–9	5.2
–36	–54	63	5.1
–34	–64	56	4.8
–14	–72	30	6.9
5	–74	23	5.9
–10	–74	2	5.7
10	–76	–4	5.9
–28	–82	14	5.5
Control eye movement minus fixation			
Coordinates			*t* value
*x*	*y*	*z*	
–53	0	–1	4.4
49	–6	–12	5.3
63	–42	8	6.8
42	–42	–15	5.7
20	–62	32	8.8
–32	–64	56	13.8
33	–64	52	12.6
27	–64	–11	12.0
–25	–68	–18	11.4
30	–88	14	11.3
–28	–88	20	11.3

## Discussion

The present fMRI study demonstrated activity related to the performance of eye movements per se in the posterior frontal cortical regions that had previously been shown to be involved in the control of eye movements, namely the FEF (FEF/sPCS) and the ventral premotor eye field (PMVe/iPCS) that are both located on the anterior precentral region, as well as the SEF on the medial frontal lobe. An additional peak that was clearly more anterior in the middle frontal gyrus was demonstrated when the subjects selected the location to look at based on previously acquired conditional rules. The latter activity that was related to the cognitive selection between competing stimuli based on previously learned conditional rules lies in the ventral part of granular area 8A on the posterior part of the middle frontal gyrus in the human brain (Petrides and Pandya, 1999 ; Fig. 6*B*). This prefrontal cortical area 8Av was here shown to be specifically involved when the subject must implement an internal cognitive rule to make a cognitive selection of the target stimulus from the alternative competing stimuli. Importantly, the activity in area 8A was not related to the performance of eye movements per se (Fig. 4*AI*,*BI*, blue vs green line plotted). Thus, area 8Av is here shown to be selectively involved in the high-level cognitive decision of which visual stimulus in the environment should be selected (i.e., attended to) at any given moment. The selected stimulus is then looked at by moving the eyes to that particular target stimulus, but the control task shows that it is not the eye movement per se that recruits this granular part of the frontal cortex, i.e., area 8A (Fig. 4*A*,*B*, green line plotted in *I* vs *II*, *III*, *IV*). This interpretation is consistent with data showing that dorsolateral frontal lesions that include area 8A in the human brain (Petrides, 1985a) and damage restricted to area 8A in the macaque monkey (Petrides, 1987) impair the ability to select between visual stimuli based on the application of conditional rules, although these subjects are not impaired in discriminating between the stimuli or moving their eyes. Thus, perceptual processing is intact, and short and long-term memory of visual stimuli is intact after lesions restricted to area 8A, but the ability to select between competing visual stimuli in the environment based on conditional rules is selectively impaired (Petrides, 1985a,b, 1986, 1987). Merriam and colleagues (2001) show that increasing cognitive demand will result in engagement of various prefrontal and parietal cortical regions adjacent to the eye fields (Merriam et al., 2001). While the illustrations provided in the publication suggest potential involvement of area 8A as some activity in the ventro-caudal middle frontal gyrus is shown, only the mid-dorsolateral peak in the rostral middle frontal gyrus is described and discussed. The mid-dorsolateral prefrontal cortex has been shown to be important for the monitoring of information in working memory (Owen et al., 1998; Petrides, 2000a,b), and this peak might be related to the fact that incompatibilities in the task might require increased monitoring.

The results of the present fMRI experiment demonstrate that the engagement of area 8Av is critical for the cognitive selection of stimuli in the environment and show a clear dissociation between area 8Av and areas posterior to it that perform eye movements per se (Amiez and Petrides, 2009; Tark and Curtis, 2009; Leoné et al., 2014; Mackey et al., 2017). The eye movement areas lie in the anterior part of the precentral motor region. By contrast, the area that is involved in the cognitive decision of which stimuli to look at (i.e., allocate attention) lies on the posterior middle frontal gyrus where granular prefrontal area 8Av is found (Petrides and Pandya, 1999).

Area 8A in the macaque monkey has been shown to be anatomically connected to multimodal cortical areas in the parietal and superior temporal sulcus, as well as the peristriate visual cortex, a set of connections that are ideal for the top-down selection of particular targets in the environment: objects or locations (i.e., targets to which attention must be allocated), and in fMRI studies, the posterior middle frontal gyrus (where area 8A lies) has been identified as a key region for top-down attentional control (Kastner and Ungerleider, 2000; Fox et al., 2006; Yeo et al., 2011; Zanto et al., 2011; Szczepanski et al., 2013; Baldauf and Desimone, 2014; Japee et al., 2015; Germann and Petrides, 2020). Area 8 neurons have been shown to exhibit an attentional bias in recorded activity when, covertly, selecting a target location in visual space (Moore et al., 2003) and microstimulation in rostral area 8 of monkeys enhances the attention bias on subsequent eye movement (Schafer and Moore, 2007). This region has also been described as a hub for visual attention in a study that examined activity in the right posterior middle frontal gyrus in association with a rule-based increase in attention to a particular stimulus (Sebastian et al., 2016). Zhang and colleagues searched for the source of the attentional bias observed throughout the visual processing cortical areas (Zhang et al., 2018). Using dynamic causal modeling of effective connectivity, they were able to conclude that the key hub for the source of the bias lies in the ventral posterior middle frontal gyrus, consistent with the present observation of activity related to the conditional selection of visual stimuli in the ventral posterior middle frontal gyrus.

The proposed role in the flexible internally guided selection of visual stimuli also explains the critical involvement of this region in task-switching and the Stroop task (Derrfuss et al., 2004, 2005; Brass et al., 2005, 2008). Derrfuss and colleagues demonstrated that this local activity associated with Stroop task performance is found rostrally to the inferior precentral sulcus eye field (PMVe/iPCS) (Derrfuss et al., 2012). Task-switching and response inhibition, both critically important during the performance of these tasks, are highly dependent on the basal ganglia (Owen et al., 1993; Robbins et al., 2012; Dalley and Robbins, 2017), and subsequent research demonstrated that the lateral prefrontal cortex is only critical in task switching when an abstract rule must be applied (Kehagia et al., 2017). Using transcranial magnetic stimulation (TMS) over area 8Av to disrupt local functioning would interfere with the subject’s ability to use internal knowledge for target selection. This is demonstrated by Muhle-Karbe and colleagues who disrupted local computations with TMS over the ventro-caudal middle frontal gyrus (coordinates *x* = 35, *y* = 12, *z* = 24) and did minimize “interference” while subjects were performing a Stroop task by eliminating interference of “internal knowledge” in target selection that in this instance was not task-relevant (Muhle-Karbe et al., 2018). Using fMRI, Kübler and colleagues show that activity in this ventro-caudal middle frontal region (peak reported as *x* = 43, *y* = 12, *z* = 32) diminishes with repetition (Kübler et al., 2006), consistent with the current proposal that it is a key region in selecting between competing targets based on cognitive rules.

Thus, the specific contribution of area 8A, which is located anterior to premotor areas involved in the selection, planning and execution of complex motor tasks, including eye movements, lies in the cognitive selection between competing stimuli in the environment via the allocation of attention. It plays a critical role in applying internal knowledge, such as goals and acquired rules, to select between alternative targets in the environment.

## Synthesis

Reviewing Editor: Satu Palva, University of Helsinki Decisions are customarily a result of the Reviewing Editor and the peer reviewers coming together and discussing their recommendations until a consensus is reached. When revisions are invited, a fact-based synthesis statement explaining their decision and outlining what is needed to prepare a revision will be listed below. Note: If this manuscript was transferred from JNeurosci and a decision was made to accept the manuscript without peer review, a brief statement to this effect will instead be what is listed below.

I have gone myself through the responses to the reviewer’s concerns and modifications to the revised manuscript and find them appropriately performed. I have no further concerns.
